# Blood pressure measurement at two years in offspring of women randomized to a trial of metformin for GDM: follow up data from the MiG trial

**DOI:** 10.1186/s12887-015-0372-1

**Published:** 2015-05-06

**Authors:** Malcolm R Battin, Victor Obolonkin, Elaine Rush, William Hague, Suzette Coat, Janet Rowan

**Affiliations:** Newborn Services, Auckland City Hospital, 9th Floor, support building, Private Bag 92 024, Auckland, New Zealand; Centre for Child Health Research, School of Sport and Recreation, Faculty of Health and Environmental Sciences, Auckland University of Technology, Auckland, New Zealand; Robinson Research Institute, University of Adelaide, Adelaide, South Australia Australia; Department of Obstetrics, National Women’s Health, Auckland City Hospital, Auckland, New Zealand

**Keywords:** Gestational diabetes, Metformin, Hypertension, Childhood blood pressure

## Abstract

**Background:**

Offspring born following maternal gestational diabetes are at risk of excessive childhood weight gain and Type 2 diabetes in childhood, which in turn is associated with an increased rate of hypertension.

We aimed to determine the systolic and diastolic blood pressure at two years of age in a cohort of children exposed to gestational diabetes mellitus using data from the MiG trial of metformin use in gestational diabetes. The secondary aim was to analyze these data by randomization of treatment to insulin or metformin.

**Methods:**

The offspring of women who had gestational diabetes and had been assigned to either open treatment with metformin (with supplemental insulin if required) or insulin in the MiG trial were followed up at 2 years of age. Oscillometric measurement of BP in the right arm was performed by a researcher using an appropriately sized cuff.

**Results:**

A total of 489 measurement blood pressure measurements were obtained in 170 of the 222 children who were seen at a median (range) age of 29 (22–38) months corrected gestational age. At the time of assessment the mean (SD) weight and height was 13.8(2) kg and 90 (4.2) cm respectively. For the whole group the mean (SD) systolic pressure was 90.9 (9.9) mmHg and mean (SD) diastolic pressure was 55.7 (8.1) mmHg. No difference was found between the metformin and insulin treatment arms. In a regression model, height and weight were only two factors associated with the levels of systolic blood pressure. For each additional kg the systolic blood pressure increased by 1.0 mmHg. For each additional cm of height the systolic blood pressure increased by 0.42 mmHg.

**Conclusions:**

Blood pressure data was obtained at approximately two years of age in a substantial cohort of children whose mothers received treatment for GDM. These novel data compare favorably with published norms.

**Clinical Trials Registry:**

This study was registered under the Australian New Zealand Clinical Trials Registry (ACTRN12605000311651).

## Background

Fetal exposure to high circulating blood glucose associated with maternal diabetes can result in major childhood effects for the offspring, particularly increased rates of obesity and Type 2 diabetes. Although these effects have been known for some time, there has been more of a focus on the topic over the last decade. Indeed, a recent review on infants born to mothers with gestational diabetes mellitus (GDM) highlighted both the increased incidence of GDM, associated with the obesity pandemic, and the potential threat to global health from long term and transgenerational effects [1].

In a landmark study of pregnancy outcome in Pima Indians, children born to mothers with Type 2 diabetes or gestational diabetes mellitus (GDM) were larger for gestational age at birth and heavier at five years of age compared with those born to non-diabetic women [2]. Subsequent studies have confirmed that exposure to maternal diabetes in-utero is associated with excessive childhood weight gain [3-5] and an increased risk of Type 2 diabetes in childhood [2,5].

Children who develop childhood Type 2 diabetes also have an increased rate of hypertension. In one surveillance report there was a six fold increase in risk, and nearly a quarter of children with Type 2 DM were found to have hypertension [6]. A combination of hypertension and childhood Type 2 diabetes has important implications for ongoing adult cardiovascular health.

Although very low birth weight and premature infants are reported to demonstrate elevated blood pressure (BP) measurements in the preschool time period [7,8] there are limited published BP data for the pre-school offspring of women with GDM [9]. Accordingly we aimed to determine the systolic and diastolic BP at two years of age in a cohort of children exposed to GDM using data from the MiG trial of metformin use in GDM [10]. The whole group data will assist with determining the contribution of GDM to child health. Although it is recognized that there is still insufficient information on the childhood effects of maternal treatment of GDM by tight control compared to minimal intervention [1], it was considered of interest to study the effect, if any, of maternal treatment type. Thus a secondary aim was to analyze these data by randomization of treatment to insulin or metformin. Body composition and anthropometric measurements for this cohort have previously been reported, with the children exposed to metformin having larger measures of subcutaneous body fat but similar total body fat to those children whose mother received insulin [11].

## Methods

The original MiG trial methods have been previously reported [10,11]. Women with GDM, whose hyperglycaemia had not responded to lifestyle measures, were randomly assigned at 20 to 33 weeks of gestation to open treatment with metformin (with supplemental insulin if required) or to insulin and a combination of neonatal and maternal outcomes were studied. The details of arrangements for follow up and body composition at 2 years of age have also been reported [11].

Women who had consented to further follow-up of their child were telephoned around the child’s second birthday to invite ongoing participation and assessment. In Auckland, a home visit was arranged and simple anthropometry measurements of the mother and child were made. A follow-up appointment was made within 1 to 2 weeks of the home visit to attend the Liggins Institute, University of Auckland, for the child to have a physical examination, including formal blood pressure measurement. In Adelaide, all but two of the follow-up assessments were performed in hospital at an outpatient research clinic, when formal blood pressure and anthropometric measurements were made. Blood samples were drawn as possible, and Bayley assessment and body composition studies were also performed, either at the same or a separate occasion.

This follow-up study had ethical approval from the Northern A Health and Disability Ethics Committee in Auckland and the Women's and Children's Hospital Network Human Research Ethics Committee in Adelaide. Written informed consent was again obtained for each participant. It ran from November 2004 to February 2008. The study was registered under the Australian New Zealand Clinical Trials Registry (ACTRN12605000311651).

BP was measured by a researcher at a morning follow up appointment using a Dinamap XL vital-signs monitor (Critikon, Tampa, FL). Measurements were made from the right arm using an appropriately sized cuff.

Data are presented as mean (SD) unless not normally distributed, when median (range) is used. Continuous data were analysed using stepwise linear regression. An initial model was obtained by fitting outcome to all covariates: gestation age, ethnicity, height, weight, waist, hip, chest and arm measurements. Factors were then removed stepwise in descending order of P-values whenever they were found to be non-significant (P < 0.05), yielding the final model. Categorical data were analysed using Chi square and Fischer’s exact test as appropriate. Box and whisker plots have been used to illustrate the data distribution.

## Results

**Figure 1:**
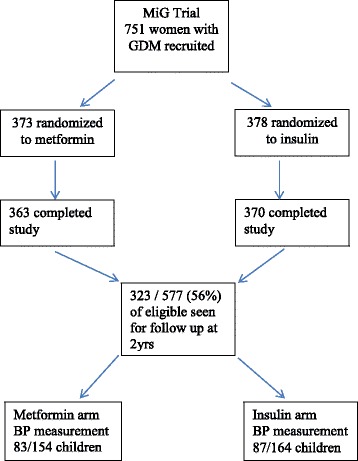
Flow diagram of follow up from original trial recruitment.

Blood pressure measurements were obtained in 170 (53%) of the total children (Figure 1) who attended for assessment at two years. The group included 89 girls (52.3%) and 81 boys (47.7%) at a median (range) age of 29 (22–38) months corrected gestational age. In the original MiG trial, the mother had been allocated to insulin for 87 (51.2%) of these children and to metformin for the remaining 83 (48.8%) children. Although over 50 percent of the group were of European ethnicity, there was a spread of ethnicity including significant numbers of Pacific and Indian children (Table 1).

**Table 1:** **Maternal Ethnicity by Initial Treatment Allocation**

**Ethnicity mother**	**Insulin-treated**	**Metformin-treated**	**Total (%)**
European	44	50	94 (55.3)
Chinese	6	6	12 (7.1)
Indian	13	7	20 (11.8)
Maori	1	4	5 (2.9)
Other	4	2	6 (3.5)
Other Asian	6	4	10 (5.9)
Polynesian	13	10	23 (13.5)
Total	87	83	170 (100)

There were no differences in distribution of treatment by ethnicity despite this being a subgroup of the original trial participants. At the time of assessment the mean (SD) weight and height was 13.8 (2.0) kg and 90.0 (4.2) cm for the whole study group. There were no differences in weight at 13.7 (1.9) kg versus 14.0 (2.0) kg and height at 90.5 (4.7) versus 90.4 (5.1) cm for insulin and metformin groups respectively. Sex was also equally distributed between the treatment groups with 41 males (47.1%) in insulin group and 40 males (48.2%) in the metformin group.

**Figure 2:**
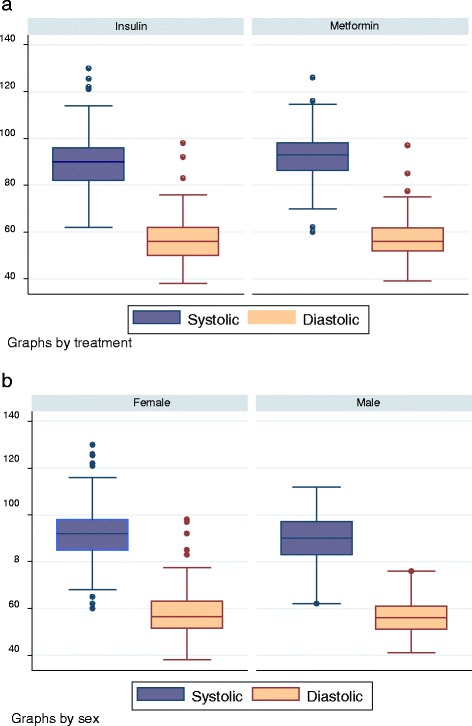
Box and whisker plot showing raw systolic and diastolic blood pressure a two years of age divided by treatment arm **(a)** and sex **(b)**.

A total of 489 measurements were recorded from 170 children (mean 2.9 per child). For the whole group the mean (SD) systolic pressure was 90.9 (9.9) mmHg and mean (SD) diastolic pressure was 55.7 (8.1) mmHg. Distribution of systolic and diastolic pressures divided by treatment group and gender are shown in the figure (Figure 2).

In the regression model, height and weight were only two significant factors associated with the systolic blood pressure. For each additional kg the systolic blood pressure increased by 1.0 mmHg (CI 0.3 to 1.7, P-value – 0.003). For each additional cm of height the systolic blood pressure increased by 0.42 mmHg (CI 0.1 to 0.7, P-value-0.003). No difference was found between the metformin and the insulin arms.

## Discussion

In this study we report data on blood pressure obtained at approximately two years of age in a substantial cohort of children whose mothers had received treatment during their pregnancy for GDM. These novel data are reassuring for the group as a whole with a mean systolic pressure of 90.9 mmHg and mean diastolic pressure of 55.7 mmHg, which compares favorably with published norms for children not exposed to GDM [8,12]. Van Houten [12] reported mean systolic and diastolic BP of 101 and 62 mmHg respectively in boys and 102 and 63 mmHg respectively in girls at a mean age of 25.1 months in a study of well children from the Netherlands. The mean weights of these Dutch children were 12.85 Kg and 12.45 Kg for males and females respectively. Our study children were slightly older at 29 months and had a slightly heavier mean weight at 14 kg but the mean length was very similar [89.6 cm and 88.7 versus 90 cm]. In another study of Australian norms, from a population reported to comprise approximately 90 percent Caucasian children [13], the 50^th^ centile for systolic/diastolic blood pressure was 99/51 with 5^th^ centile 87/39 and 95^th^ 114/64 mmHg at 3 years.

In a study of infants from the USA who were exposed to GDM, the mean systolic BP at 3 years of age was reported to be 3.2 mmHg higher than that in controls born to normoglycaemic mothers after adjustment for sex age and measurement condition. In this study population, of whom 73% were born to mothers described as white American, the mean systolic BP was 92 mmHg, which is similar to the US 50^th^ centile [9]. A recent systematic review [14] has reported increased systolic BP following exposure to diabetes in-utero but no effect on diastolic BP. However, there were important differences in type of maternal diabetes, sex and age at follow up that require further investigation. Two years of age is an early time point to observe BP, so the MiG cohort are currently being followed up and having their BP measured in later childhood, with plans to continue surveillance into adolescence and adulthood. In a very small group of 25 children followed up following in utero metformin exposure given for maternal polycystic ovarian syndrome a small but significant increase in systolic blood pressure was reported [15]. The authors correctly pointed out that, given the small numbers in the study, further research was required before conclusions could be made. On univariate analysis of our study data, there was a small difference in raw systolic blood pressure (mean 89.8 mmHg versus 92.2 mmHg) between the arms, but this was not significant in the stepwise linear regression model that adjusted for gestational age, height, weight and arm measurements.

The methodology for the study is robust in that the BP data were prospectively collected by dedicated researchers, all highly motivated and who had time to settle the child and achieve a co-operative state prior to performing the measurements. Attempts were made to collect more than one recording. In addition, treatment allocation was subject to the usual care involved in a randomized controlled trial.

The two study limitations of note were first that, as mentioned above, two years of age is early in childhood to detect significant differences in cardiovascular health, and second, that BP measurement was only obtained from the subgroup of the offspring (53%) who returned to the follow up appointment. The rate of successfully measuring blood pressure in our cohort is comparable with that published by Van Houten et al. [12], who reported successful measurement in 63 percent of 836 children at two years of age. However, it represents an overall follow up of just under 30 percent of the 577 children potentially available from the original MiG trial [10]. A low follow up rate could introduce variation from the original study, so it was important to examine the characteristics of the group. Except for a lower proportion of Pacific Island ethnicity, however, this group were not different in baseline characteristics from those in the initial trial [10].

## Conclusion

It is recognized that adverse perinatal events, including prematurity, may be associated with elevated blood pressure in childhood [7]. An increase in childhood systolic, but not diastolic, blood pressure has been reported in offspring following maternal diabetes in pregnancy [14]. The offspring of pregnant women who develop GDM are also at an increased risk of developing type 2 diabetes. In this follow up of offspring from women with GDM studied in the MiG trial the systolic blood pressure was comparable with reported norms and showed no difference between treatment arms. Although it is important to continue to assess the cardiovascular status of this group as they proceed through childhood, the current study provides important novel data at two years of age for children exposed to the in utero effects of GDM.
